# Supplementary material for: Validation of the performance of a point of care molecular test for leprosy: From a simplified DNA extraction protocol to a portable qPCR
Source: PLoS Negl Trop Dis. 2024 Oct 7;18(10):e0012032. doi: 10.1371/journal.pntd.0012032 (PMC11573133; doi:10.1371/journal.pntd.0012032)
Supplement: S3 Table — (DOCX) [file pntd.0012032.s003.docx]

| **TITLE OR ABSTRACT** |  |
| --- | --- |
| **1** | **Identification as a study of diagnostic accuracy using at least one measure of accuracy (such as sensitivity, specificity, predictive values, or AUC)**  Development and validation of a simplified DNA extraction protocol suitable for a coupled to a portable device (Q3-Plus) qPCR to detection of Mycobacterium leprae in low-infrastructure settings |
| **ABSTRACT** |  |
| **2** | **Structured summary of study design, methods, results, and conclusions (for specific guidance, see STARD for Abstracts)**  The study aimed to optimize qPCR reactions using oligonucleotides from the first Brazilian molecular diagnostic kit for leprosy on a portable platform (Q3-Plus). In addition, we sought to develop a simplified protocol for DNA extraction that met point-of-care criteria. During optimization on the Q3-Plus, optical parameters, thresholds, and cutoffs for the *16S rRNA* and RLEP targets of *M. leprae* were established using synthetic DNA, purified DNA from *M. leprae*, and pre-characterized clinical samples. In the simplified extraction step, different lysis solutions were evaluated using chaotropic agents, and purification was carried out by depositing the lysed material on FTA cards. The complete protocol (simplified extraction + qPCR on the portable platform) was evaluated with pre-characterized clinical skin biopsy samples and compared with standard equipment (QuantStudio-5). LOD_95%_ for the optimized reactions was 113.31 genome-equivalents/μL for *16S rRNA* and 17.70 genome-equivalents/μL for RLEP. Among the lysis solutions, the best-performing was composed of urea (2 M), which provided good dissolution of the skin fragment and a lower Ct value, indicating higher concentrations of DNA. The complete technological solution showed a sensitivity of 52% in reactions. Our results highlight the need for additional optimization to deal with paucibacillary samples, but also demonstrate the applicability of the portable platform in the detection of *M.* leprae in low infrastructure settings. |
| **INTRODUCTION** |  |
| **3** | **Scientific and clinical background, including the intended use and clinical role of the index test**  Leprosy is a chronic and progressive disease caused by *Mycobacterium leprae.* Due to the various clinical forms associated with this condition, its diagnosis becomes complex. In this regard, clinical symptom evaluation is primarily employed by healthcare professionals to establish a diagnosis. Bacilloscopic analysis is also commonly used as an auxiliary method; however, it lacks sensitivity, as do indirect serological methods. The absence of a gold-standard method with high sensitivity and specificity impacts diagnostic accuracy and promotes disease progression and transmission. This study aimed to optimize qPCR reactions using a commercial kit for *M. leprae* detection in a portable device, facilitating population screening in remote areas. |
| **4** | **Study objectives and hypotheses**  The aimed of the study was to optimize qPCR reactions utilizing the NAT Hans kit on the portable Q3-Plus equipment. Simultaneously, we aimed to develop a simplified DNA extraction protocol for *M. leprae*. This would enable the implementation of a leprosy screening test in remote areas. |
| **METHODS** |  |
| *Study design*  **5** | **Whether data collection was planned before the index test and reference standard were performed (prospective study) or after (retrospective study)**  The samples were collected based on the occurrence of suspected cases of leprosy and other dermatoses at Souza Araujo clinic. Were performed a prospective study. |
| *Participants*  **6** | **Eligibility criteria**  The patients were selected according to the occurrence of suspected cases of leprosy at the Souza Araujo clinic. |
| **7** | **On what basis potentially eligible participants were identified (such as symptoms, results from previous tests, inclusion in registry)**  Participants potentially included in the study were selected in the screening service at the Souza Araújo outpatient clinic because they had skin lesions suspected of leprosy or other dermatoses. |
| **8** | **Where and when potentially eligible participants were identified (setting, location and dates)**  All participants were selected and identified at the Souza Araújo outpatient clinic, Fiocruz- Rio de Janeiro throughout the year 2023. |
| **9** | **Whether participants formed a consecutive, random or convenience series**  Study participants were selected for convenience |
| *Test methods*  **10a** | **Index test, in sufficient detail to allow replication**  The index test is an analysis of the accuracy of the portable qPCR Q3-Plus equipment for molecular diagnosis of leprosy.  We tested in sufficient detail to allow replication |
| **10b** | **Reference standard, in sufficient detail to allow replication**  In the case of molecular diagnosis of leprosy, the reference standard is analysis using traditional real-time qPCR equipment (7500 Standard and Q3-plus equipments). |
| **11** | **Rationale for choosing the reference standard (if alternatives exist)**  We use the real-time PCR equipment we have in our laboratory |
| **12a** | **Definition of and rationale for test positivity cut-offs or result categories of the index test, distinguishing pre-specified from exploratory**  The commercial NAT Hans kit (IBMP) served as the reference standard in the paired analyses between the Quantstudio 5 and Q3-Plus devices. This registered kit comes with pre-established cut-off parameters. To establish the cut-off values on the Q3-Plus equipment, a Bland-Altman analyses was conducted, and the average Ct variation for the *16S rRNA* and RLEP targets were added to the Ct values predefined by the NAT Hans kit. |
| **12b** | **Definition of and rationale for test positivity cut-offs or result categories of the reference standard, distinguishing pre-specified from exploratory**  The commercial NAT Hans kit (IBMP) served as the reference standard in the paired analyses between the Quantstudio 5 and Q3-Plus devices. This registered kit comes with pre-established cut-off parameters. To establish the cut-off values on the Q3-Plus equipment, a Bland-Altman analyses was conducted, and the average Ct variation for the *16S rRNA* and RLEP targets were added to the Ct values predefined by the NAT Hans kit. |
| **13a** | **Whether clinical information and reference standard results were available to the performers/readers of the index test**  Yes. |
| **13b** | **Whether clinical information and index test results were available to the assessors of the reference standard**  Yes. |
| *Analysis*  **14** | **Methods for estimating or comparing measures of diagnostic accuracy**  Accuracy = (True Positive + True Negative) / (True Positive + False Positive + False Negative + True Negative). (link: https://aps.bvs.br/apps/calculadoras/?page=1) |
| **15** | **How indeterminate index test or reference standard results were handled**  Samples identified as indeterminate in the replicates were classified as such. Since this screening test, it is essential to complement it with other methods that can assist in diagnosis, such as the gold standard based on clinical symptomatology. |
| **16** | **How missing data on the index test and reference standard were handled**  Samples with missing data were excluded from the analyses. |
| **17** | **Any analyses of variability in diagnostic accuracy, distinguishing pre-specified from exploratory**  No |
| **18** | **Intended sample size and how it was determined**  The sample number was calculated based on the demand of suspected leprosy`s patients attended per year at the screening consultation at the Souza Araujo outpatient clinic. |
| **RESULTS** |  |
| *Participants*  **19** | **Flow of participants, using a diagram**  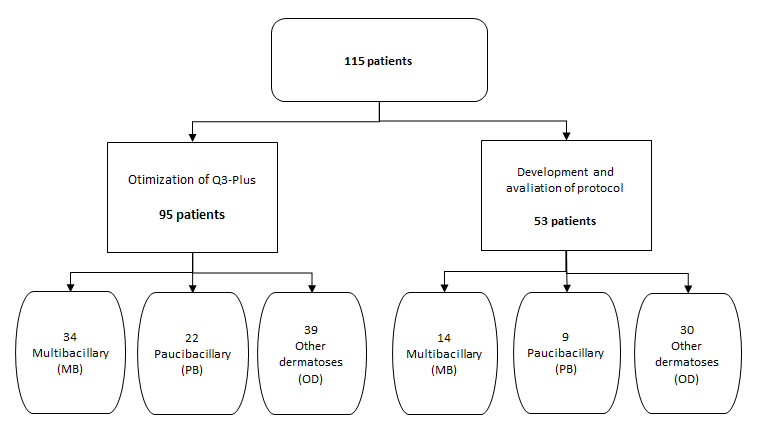 |
| **20** | **Baseline demographic and clinical characteristics of participants**  Among the total of 115 study participants, 62 study participants were men and 53 women. Two individuals were under 15 years old; sixteen were between 16 and 30 years old; thirty-one were between 31 and 45 years old; thirty-two between 46 and 60 years old and thirty-four individuals were over 60 years old.  Clinical characteristics of participants:  Paucibacillary leprosy (PB): 22% (25/115), Multibacillary leprosy (MB): 35% (40/115), patients with other dermatoses disease (ODD): 43% (50/115) |
| **21a** | **Distribution of severity of disease in those with the target condition**  Paucibacillary leprosy (PB): 25 patients  Multibacillary leprosy (MB): 40 patients |
| **21b** | **Distribution of alternative diagnoses in those without the target condition**    All patients had clinical, bacilloscopic and histopathological diagnoses. |
| **22** | **Time interval and any clinical interventions between index test and reference standard**  Patient samples were analyzed by both qPCR systems (portable and standard) at the same time. |
| *Test results*  23 | **Cross tabulation of the index test results (or their distribution) by the results of the reference standard**   \|  \| **Q3** \| **QS-5** \| \| --- \| --- \| --- \| \| **Positive (MB/PB)** \| 11 \| 13 \| \| **Negative (OD)** \| 20 \| 27 \| \| **Indeterminate** \| 10 \| 1 \| |
| **24** | **Estimates of diagnostic accuracy and their precision (such as 95% confidence intervals)**  Accuracy = (True Positive + True Negative) / (True Positive + False Positive + False Negative + True Negative). (link: <https://aps.bvs.br/apps/calculadoras/?page=1>) |
| **25** | **Any adverse events from performing the index test or the reference standard**  No adverse events occurred during this study from performing the index test or the reference standard |
| **DISCUSSION** |  |
| **26** | **Study limitations, including sources of potential bias, statistical uncertainty, and generalizability**  As a limitation of this study, it should be considered that the samples were collected based on their occurrence in the clinic. The final clinical outcome of cases will be determined after one year of follow-up. Additionally, molecular test positivity can also occur in cases under treatment, where residual bacillus DNA may be present. |
| **27** | **Implications for practice, including the intended use and clinical role of the index test**  The aim of the test is to improve access to molecular tests for the detection of the *M. leprae* agent in remote regions, enabling the screening and identification of positive and suspected cases by healthcare professionals. |
| **OTHER INFORMATION** |  |
| **28** | **Registration number and name of registry**  Not applicable |
| **29** | **Where the full study protocol can be accessed**  The complete study protocol is presented in this article |
| **30** | **Sources of funding and other support; role of funders**  FAPERJ, CNPq, Ministério da Saúde, CAPES. |
