# Supplementary material for: Validation of the performance of a point of care molecular test for leprosy: From a simplified DNA extraction protocol to a portable qPCR
Source: PLoS Negl Trop Dis. 2024 Oct 7;18(10):e0012032. doi: 10.1371/journal.pntd.0012032 (PMC11573133; doi:10.1371/journal.pntd.0012032)
Supplement: S4 Table — (DOCX) [file pntd.0012032.s004.docx]

**Supplemental Table S2. MIQE checklist.**

| **ITEM TO CHECK** | **IMPORTANCE** | **CHECKLIST** |
| --- | --- | --- |
| **EXPERIMENTAL DESIGN** |  |  |
| Definition of experimental and control groups | **E** | 1. Experimental groups: 115 samples (41 multibacillary leprosy patients, 25 paucibacillary leprosy patients and 49 other dermatoses) 2. Control groups: Extracted commercial DNA; Synthetic double strand DNA; *Mycobacterium leprae* cells; 115 pre-characterized samples (41 multibacillary, 25 paucibacillary and 49 other dermatoses) |
| Number within each group | **E** | Yes (see above) |
| Assay carried out by core lab or investigator's lab? | D | Investigator's lab |
| Acknowledgement of authors' contributions | D | Yes |
| **SAMPLE** |  |  |
| Description | **E** | Skin biopsy samples |
| Volume/mass of sample processed | D | 3 mm collected using surgical punch |
| Microdissection or macrodissection | **E** | Not performed. |
| Processing procedure | **E** | Skin Biopsy stored in 70% ethanol until sample processing. |
| If frozen - how and how quickly? | **E** | Samples were not frozen. |
| If fixed - with what, how quickly? | **E** | Samples were not fixed. |
| Sample storage conditions and duration (especially for FFPE samples) | **E** | Samples were stored in refrigerator until DNA extraction. Extracted DNA was kept in -20 ^o^C freezer. |
| **NUCLEIC ACID EXTRACTION** |  |  |
| Procedure and/or instrumentation | **E** | Silica-based spin columns and **simplified DNA extraction protocol (urea solution).** |
| Name of kit and details of any modifications | **E** | DNAeasy Blood and Tissue (Qiagen, Germany). |
| Source of additional reagents used | D |  |
| Details of DNase or RNAse treatment | **E** | Not performed. |
| Contamination assessment (DNA or RNA) | **E** | Contamination by proteins was assessed spectrophotometrically. |
| Nucleic acid quantification | **E** | Yes |
| Instrument and method | **E** | Nanodrop 2000c; spectrometry |
| Purity (A260/A280) | D | Not reported. |
| Yield | D | Not reported. |
| RNA integrity method/instrument | **E** | Not applicable. |
| RIN/RQI or Cq of 3' and 5' transcripts | **E** | Not applicable. |
| Electrophoresis traces | D | Not applicable. |
| Inhibition testing (Cq dilutions, spike or other) | **E** | Cq values for the human control target out of specified ranges were interpreted as inhibition of the reaction or failure of DNA extraction, and samples DNA were re-extracted. Non-template controls (NTC, molecular grade water) as well as leprosy-negative human DNA were routinely used as controls. |
| **REVERSE TRANSCRIPTION** |  |  |
| Complete reaction conditions | **E** | Not applicable. |
| Amount of RNA and reaction volume | **E** | Not applicable. |
| Priming oligonucleotide (if using GSP) and concentration | **E** | Not applicable. |
| Reverse transcriptase and concentration | **E** | Not applicable. |
| Temperature and time | **E** | Not applicable. |
| Manufacturer of reagents and catalogue numbers | D | Not applicable. |
| Cqs with and without RT | D* | Not applicable. |
| Storage conditions of cDNA | D | Not applicable. |
| **qPCR TARGET INFORMATION** |  |  |
| If multiplex, efficiency and LOD of each assay. | **E** | **Synthetic DNA** *- Mycobaterium leprae* DNA detection (**Q3-Plus)**: *16S rRNA* target – efficiency of 109% and LOD95% 13.86 copies per microliter. RLEP target – efficiency of 108% and LOD95% 13.86 copies per microliter.  ***M.leprae* cells -** *16S rRNA* target – efficiency of 131% and LOD95% 113.31 genome equivalents per microliter. RLEP target – efficiency of 105% and LOD95% 17.70 genome equivalents per microliter. |
| Sequence accession number | **E** | Not applicable. |
| Location of amplicon | D | Not applicable. |
| Amplicon length | **E** | Not applicable. |
| *In silico* specificity screen (BLAST, etc) | **E** | Not applicable. |
| Pseudogenes, retropseudogenes or other homologs? | D | -- |
| Sequence alignment | D | Not applicable. |
| Secondary structure analysis of amplicon | D | -- |
| Location of each primer by exon or intron (if applicable) | **E** | Not performed. |
| What splice variants are targeted? | **E** | Not performed. |
| **qPCR OLIGONUCLEOTIDES** |  |  |
| Primer sequences | **E** | *16S rRNA* – foward: 5´-GCATGTCTTGTGGTGGAAAGC- 3´  Reverse: 5´-CACCCCACCAACAAGCTGAT- 3´  RLEP – forward: : 5´-GCAGCAGTATCGTGTTAGTGAA-3´  Reverse: 5´-CGCTAGAAGGTTGCCGTAT-3´  *18S rRNA* – forward: 5´-GAAACTGCGAATGGCTCATTAAATCA- 3´  Reverse: 5´-CCCGTCGGCATGTATTAGCTCT-3´ |
| RTPrimerDB Identification Number | D | -- |
| Probe sequences | D** | 16S rRNA - 5´-CATCCTGCACCGCA-3´  RLEP - 5´CGCCGACGGCCGGATCATCGA-3´  18S rRNA - 5´GGAGCGAGCGACCAAAGGAACCA-3´ |
| Location and identity of any modifications | **E** | Not applicable. |
| Manufacturer of oligonucleotides | D | Exxtend (São Paulo, Brazil) |
| Purification method | D | reverse phase and HPLC |
| **qPCR PROTOCOL** |  |  |
| Complete reaction conditions | **E** | Detection of *Mycobacterium leprae* and human 18S rRNA gene DNA was performed using the oligonucleotides developed by MANTA et al. (2022). GoTaq Probe qPCR Master Mix (2x) (Promega, USA), 2 µL of extracted DNA, oligonucleotides *16S rRNA*F (0.75 µM), *16S rRNA*R (0.75 µM), *16S rRNA*P (0.3 µM), RLEPF (0.4 µM), RLEPR (0.4 µM), and RLEPP (0.2 µM) and *18S rRNA*F (0.2 µM), *18S rRNA*R (0.2 µM) and *18S rRNA*P (0.1 µM) in a reaction volume of 5 µL (Q3-Plus) |
| Reaction volume and amount of cDNA/DNA | **E** | Reaction volume of 5 µL and 2 µL of extracted DNA. |
| Primer, (probe), Mg++ and dNTP concentrations | **E** | *16S rRNA*F and *16S rRNA*R: 0.75 µM; *16S rRNA*P: 0.3 µM  RLEPF and RLEPR: 0.4 µM; RLEPP: 0.2 µM  *18S rRNA*F and *18S rRNA*R: 0.2 µM ; *18S rRNA*P: 0.1 µM  GoTaq Probe qPCR Master Mix (2X) (Promega, USA). |
| Polymerase identity and concentration | **E** | Taq DNA polymerase  (GoTaq Probe qPCR Master Mix (2X) (Promega, USA). |
| Buffer/kit identity and manufacturer | **E** | GoTaq Probe qPCR Master Mix (2X) (Promega, USA) |
| Exact chemical constitution of the buffer | D | -- |
| Additives (SYBR Green I, DMSO, etc.) | **E** | None used. |
| Manufacturer of plates/tubes and catalog number | D | -- |
| Complete thermocycling parameters | **E** | Q3-Plus: 95˚C/2 min, and 45 x [95˚C/15 sec + 64˚C/1 min], using ROX as passive fluorescence. Baseline was automatically defined by the equipment for both targets. For *M.leprae 16S rRNA* target, threshold was set to 36 a.u., and cutoff value 36.9 quantification cycle (Cq). For RLEP, threshold was set to 150 a.u. and cutoff of Cq values were 39.6. For human target (18S rRNA), threshold was set to 21 a.u.. |
| Reaction setup (manual/robotic) | D | Manual |
| Manufacturer of qPCR instrument | **E** | **Q3-Plus** – STMicroeletronics - Italy |
| **qPCR VALIDATION** |  |  |
| Evidence of optimisation (from gradients) | D | -- |
| Specificity (gel, sequence, melt, or digest) | **E** | qPCR was validated using samples previously categorized by a distinct qPCR assay (instrument). |
| For SYBR Green I, Cq of the NTC | **E** | Not applicable. |
| Standard curves with slope and y-intercept | **E** | **Synthetic DNA** *- M.leprae* targets detection **(Q3-Plus)**: *16S rRNA* slope -3.12, Y-intercept of +37.08 and RLEP slope -3.15, Y- intercept of +42.06.  ***M.leprae* cells**  *16S rRNA* slope -2.75, Y-intercept of +49.16 and RLEP slope -3.22, Y-intercept of +51.82. |
| PCR efficiency calculated from slope | **E** | **Synthetic DNA**  *16S rRNA* efficiency of 109%, and RLEP efficiency of 108%.  ***M.leprae* cells**  *16S rRNA* efficiency 131%, and RLEP efficiency of 105%. |
| Confidence interval for PCR efficiency or standard error | D | Not shown. |
| r2 of standard curve | **E** | **Q3-Plus**: Synthetic DNA= 16S rRNA 98.17% ; RLEP 98.13%.  *M.leprae* cells = 16S rRNA 98.19%; RLEP 95.85% |
| Linear dynamic range | **E** | 10^5^ to 10^1^ copies/µL synthetic DNA  10^6^ to 10^2^ equivalent genome/µL of *M.leprae* |
| Cq variation at lower limit | **E** | *16S rRNA* SD of 0.47 Ct at lower limit (10 copies/µL)  RLEP SD of 0.89 Ct at lower limit (10 copies/µL) |
| Confidence intervals throughout range | D | Not shown. |
| Evidence for limit of detection | **E** | Probit calculations, described in Methods and Results. |
| If multiplex, efficiency and LOD of each assay. | **E** | **Synthetic DNA** *- M.leprae* DNA detection **(Q3-Plus):** 16S efficiency of 109% (slope –3.12) and LOD95% of 13.04 copies/µL; RLEP efficiency of 108% (slope -3.15) and LOD95% of 13.04 copies/µL.  ***M.leprae*** **cells** – (**Q3-Plus):** 16S efficiency 131% (slope -2.75) and LOD95% of 113.31 genome-equivalents/µL; RLEP efficiency of 105% (slope -3.22) and LOD95% 17.70 genome-equivalents/µL. |
| **DATA ANALYSIS** |  |  |
| qPCR analysis program (source, version) | **E** | Q3-Plus V2 Suit, version 4.0, ST Microeletronics. |
| Cq method determination | **E** | **Q3-Plus:** Baseline was set automatically for both targets. For 16S rRNA, threshold was set 36 and for RLEP threshold was set to 150. For human DNA, threshold was set to 21. |
| Outlier identification and disposition | **E** | For M.leprae DNA, quantification cycle (Cq) values below 36.5 in the 16S rRNA target were considered positive. For the RLEP target, detections below 39.5 were considered positive. |
| Results of NTCs | **E** | Non-template controls should show no amplification for both targets, whereas leprosy-negative controls should show positive detections for human DNA. |
| Justification of number and choice of reference genes | **E** | *18S rRNA* is a common reference gene for human samples. |
| Description of normalisation method | **E** | ROX as passive fluorescence |
| Number and concordance of biological replicates | D | -- |
| Number and stage (RT or qPCR) of technical replicates | **E** | All qPCR assays in Q3-Plus system were performed in technical duplicates, except for LOD determination (9-10 replicates). |
| Repeatability (intra-assay variation) | E | Repeatability to ***16S rRNA*** was found to be <9.23% (highest at 10^0 copies/µL) when assayed by three independent experienced operators.  Repeatability to **RLEP** was found to be <5.10% (highest at 10^0 copies/µL) when assayed by three independent experienced operators. |
| Reproducibility (inter-assay variation, %CV) | D | Reproducibility (inter-assay variation) to ***16S rRNA*** was found to be <3.09% (highest at 10^0 copies/µL) when assayed by three independent experienced operators.  Reproducibility (inter-assay variation) to **RLEP** was found to be <3.10% (highest at 10^0 copies/µL) when assayed by three independent experienced operators. |
| Power analysis | D | Not performed. |
| Statistical methods for result significance | **E** | Analysis of variance (ANOVA) was used to evaluate the difference between results obtained in Repeatability and Reproducibility in Q3-Plus instrument. |
| Software (source, version) | E | R scripts |
| Cq or raw data submission using RDML | **D** | -- |
