# Supplementary figures and images for: Validation of the performance of a point of care molecular test for leprosy: From a simplified DNA extraction protocol to a portable qPCR
Source: PLoS Negl Trop Dis. 2024 Oct 7;18(10):e0012032. doi: 10.1371/journal.pntd.0012032 (PMC11573133; doi:10.1371/journal.pntd.0012032)

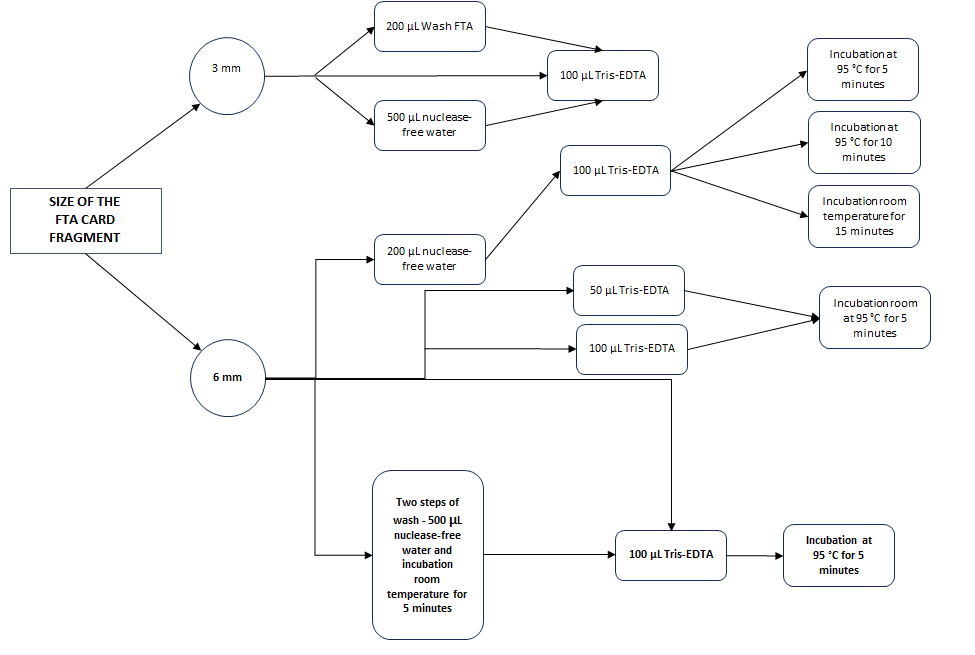

Supplement: S1 Fig — (Created by the author) (TIF) [file pntd.0012032.s010.tif]

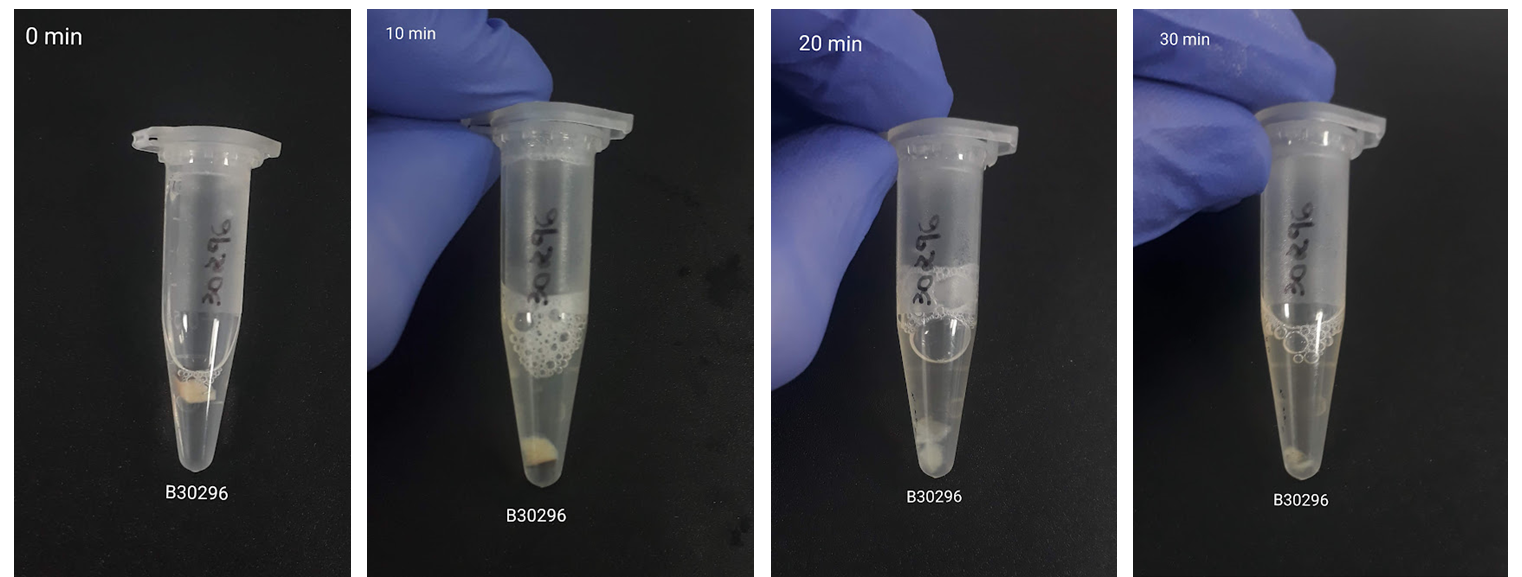

Supplement: S2 Fig — After each 10-minute step, a reduction in skin fragments and a change in the turbidity of the solution were observed. (Created by the author) (TIF) [file pntd.0012032.s011.tif]

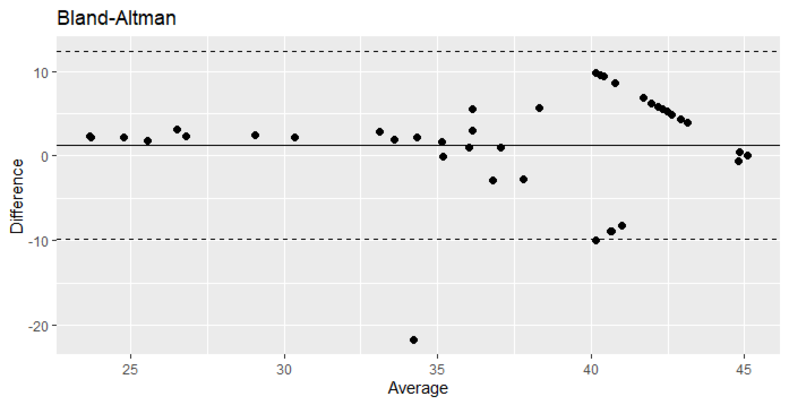

Supplement: S3 Fig — Bland-Altman analysis in 16S rRNA target. The mean difference was 1.29 cycle of threshold between the equipment in 95% confidence interval. The upper limit of agreement was 12.39 and the lower limit of agreement was -9.81. (Created by the author) (TIF) [file pntd.0012032.s012.tif]

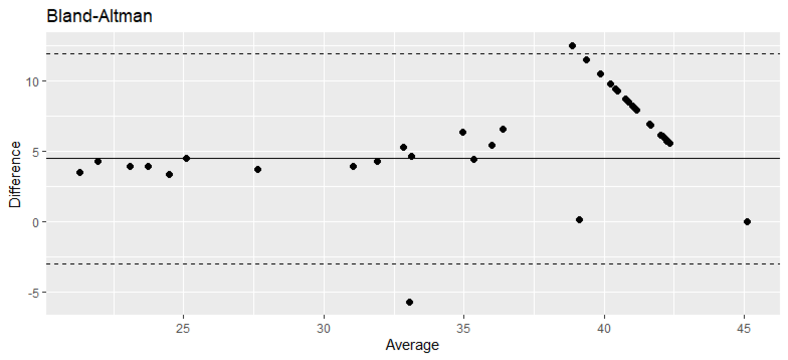

Supplement: S4 Fig — Bland-Altman analysis in RLEP target. The mean difference was 4.44 cycle of threshold between the equipment in 95% confidence interval. The upper limit of agreement was 11.91 and the lower limit of agreement was -3.04. (Created by the author) (TIF) [file pntd.0012032.s013.tif]

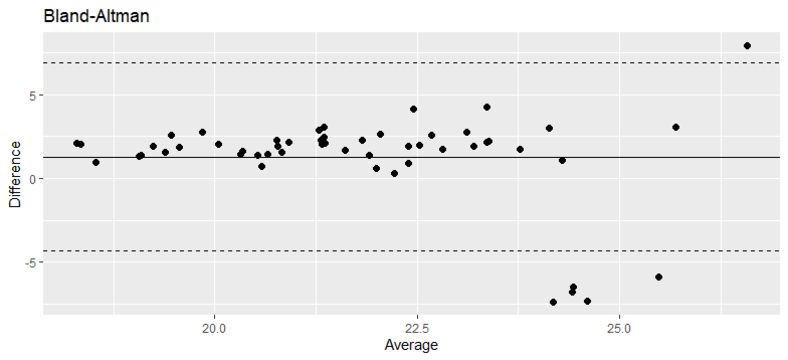

Supplement: S5 Fig — Bland-Altman analysis in 18S rRNA target. The mean difference was 1.34 cycle of threshold between the equipment in 95% confidence interval. The upper limit of agreement was 6.90 and the lower limit of agreement was -4.22. (Created by the author) (TIF) [file pntd.0012032.s014.tif]

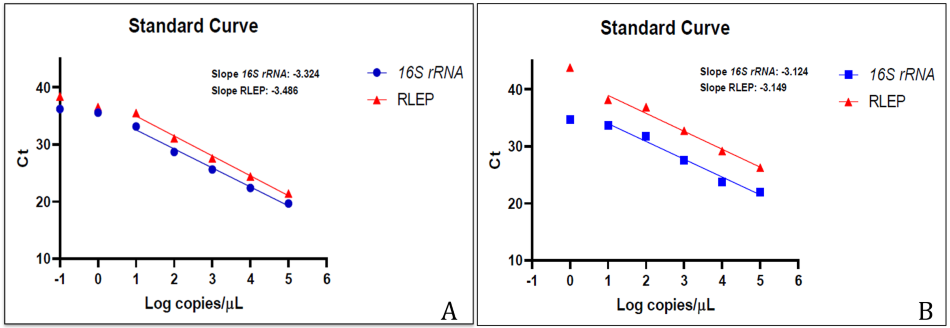

Supplement: S6 Fig — (A) Reactions on QuantStudio 5; (B) Reactions on portable platform Q3-Plus. Linear regressions were obtained from no less than 4 independent experiments. (Created by the author) (TIF) [file pntd.0012032.s015.tif]

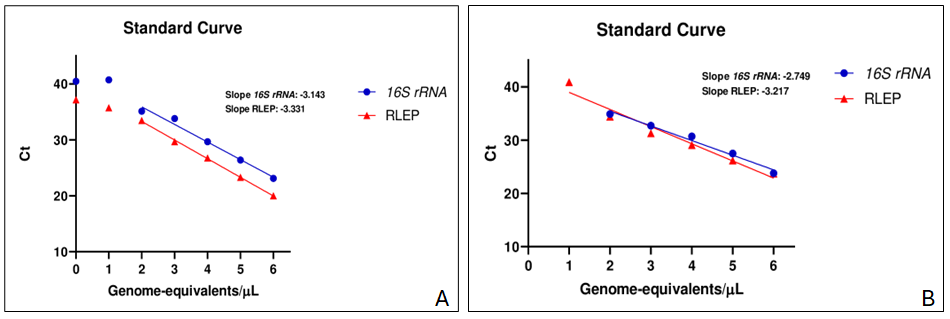

Supplement: S7 Fig — (A) Reactions on QuantStudio 5; (B) Reations on portable platform Q3-Plus. Linear regressions were obtained from no less than 4 independent experiments. (Created by the author) (TIF) [file pntd.0012032.s016.tif]

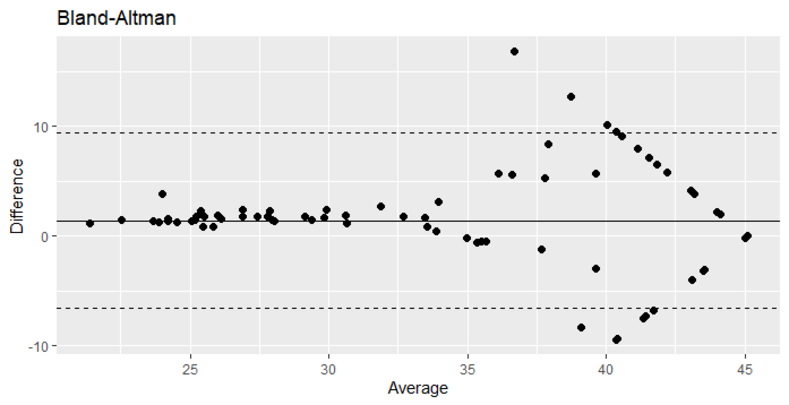

Supplement: S8 Fig — The mean difference it was 1.40 cycle of threshold between instruments. The upper limit of agreement with 95% confidence interval was 9.40 and the lower limit of agreement was -6.61. (Created by the author) (TIF) [file pntd.0012032.s017.tif]

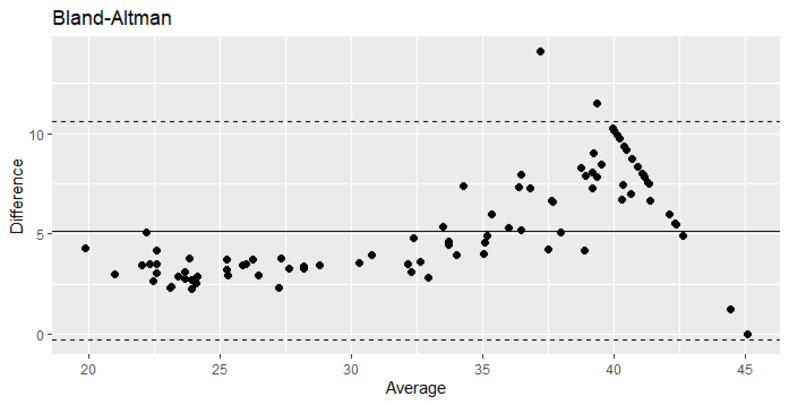

Supplement: S9 Fig — The mean difference it was 5.15 cycle of threshold between the equipment in 95% confidence interval. The upper limit of agreement was 10.59 and the lower limit of agreement was -0.28. (Created by the author) (TIF) [file pntd.0012032.s018.tif]

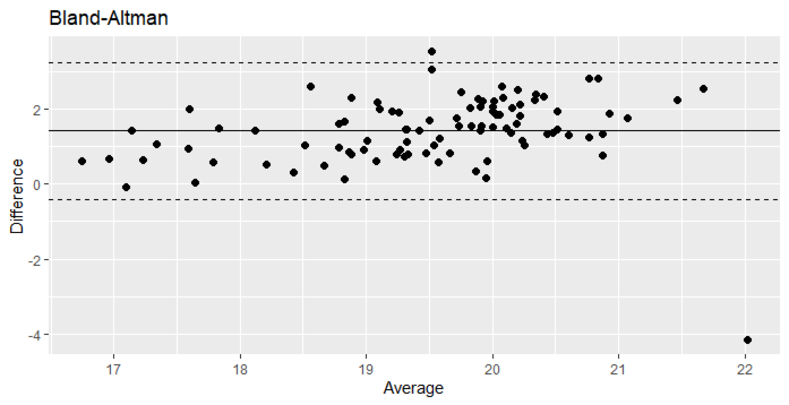

Supplement: S10 Fig — The mean difference it was 1.42 cycle of threshold between the equipment in 95% confidence interval. The upper limit of agreement was 3.25 and the lower limit of agreement was -0.40. (Created by the author) (TIF) [file pntd.0012032.s019.tif]
