# Supplementary material for: Validation of the performance of a point of care molecular test for leprosy: From a simplified DNA extraction protocol to a portable qPCR
Source: PLoS Negl Trop Dis. 2024 Oct 7;18(10):e0012032. doi: 10.1371/journal.pntd.0012032 (PMC11573133; doi:10.1371/journal.pntd.0012032)
Supplement: Attachment — Submitted filename: Reply to reviewers_27082024.docx [file pntd.0012032.s020.docx]

**Point by point reply to reviewer’s comments**

**In general:**

1. To evaluate M. leprae DNA extraction methods, authors should extract from a known quantity of *M. leprae* cells and show a comparison of qPCR data for the different conditions (i.e. lysis buffers) for each of the *M. leprae* targets.

**Answer:** The reviewer raises a very important point indeed, but a very difficult one to execute. We would like to highlight some inherent limitations regarding leprosy: *Mycobacterium leprae* does not grow *in vitro*, which complicates the maintenance and access to samples with a specified number of cells. Laboratory maintenance of the bacterium is performed in mouse footpads, which ideally would be used for the feasibility of the experiment suggested, but despite several attempts our research group currently lacks access to such samples. Other materials such as blood or intradermal scrapings are not suitable since the protocol presented was designed for skin biopsies. An alternative could be using biopsies from patients; however, this is also limiting as obtaining multiple biopsies from the same patient is unlikely to be authorized due to the invasive procedure.

To overcome these limitations and achieve reliable results, we used commercial pig skin for our experiments, which is routinely used as a substitute for human skin. We conducted all necessary replicates using this tissue to evaluate different DNA extraction methods and to detect a constitutively expressed gene, which is a marker of the extraction efficiency of each method.

2. To evaluate the accuracy of the new test, authors should report Ct scores for each separate group (MB, PB, and OD) for both of the *M. leprae* targets.

**Answer:** Thanks for the suggestion. Ct values for each group (MB, PB and OD) are now included as supplementary tables (S5 TABLE and S9 TABLE). Performance data were reassessed and incorporated in the text (lines 378 and 459) and Tables 2 and 3.

**Methods:**

**Reviewer 1 (pink):** Are there concerns about ethical or regulatory requirements being met?

Yes, although not clear where the pork skin comes from and whether ethical approval is necessary in Brazil for this .

**Answer:** The pork skin used in this study was acquired from local butchers. No live animals were used at any stage of the research process. In Brazil, no ethical approval is necessary for this type of study.

**Reviewer 2 (green):** The objectives of the study are articulated well although the organization and flow could be improved. The study design involves MB and PB leprosy patients and other dermatoses (OD) but the numbers in these groups change with different extraction protocols so it is a little hard to follow.

**Answer:** Thanks for the suggestion. We now present a new FIGURE 1, displaying a flowchart of all analyses carried out during this study, detailing the number and type of samples used in each experiment. For optimization of qPCR reactions on the portable instrument we used 95 clinical samples, of which 34 were multibacillary (MB), 22 were paucibacillary (PB), and 39 were other dermatoses (OD). For the optimization of the simplified extraction DNA protocol, we used 53 clinical samples, of which 14 were MB, 9 were PB , and 30 were OD (line 160 and 162).

**Reviewer 3 (blue):** The major comments are the following: the methods are far too detailed for a publication and all the intermediary steps to reach the final protocol need to be deleted or put in an appendix or supplementary materials (e.g. tables and 1 and 2). only the method finally approved and that was used to compare the results, need to be described herein.

**Answer:** Thank you for raising this point. We have carefully considered your suggestions and have made revisions to the article accordingly. Tables 1 and 2 were moved to supplementary materials.

**Results**

**Reviewer 1:** Figures lack abbreviation explanations. Results can be more concise and better structured.

**Answer:** Thanks for the feedback. Explanations for abbreviations in all figures have been included. We have considered your suggestions and inserted a flowchart as a summary of the analysis carried out (Figure 1), in addition to redirecting some tables to supplementary materials.

**Reviewer 2:** The results for the three groups, MB and PB patients and OD are not presented well, there is no clear cut comparison of the sensitivity and specificity between the MB, PB patients and the OD group.

**Answer:** Samples were separated according to the clinical form for sensitivity and specificity analyses. Results were included in Tables 2 and 3 and updated in the manuscript (lines 378 and 459).

**Reviewer 2:** There should also be an evaluation shown of all six extraction buffers with a known amount of *M. leprae* whole cells from nude mouse footpad with the Ct scores shown to show which extraction solution is best.

**Answer:** We understand the importance of this evaluation and agree it would be the best approach. However, due to difficulties in accessing this type of sample, we conducted the analysis using a porcine skin model. To standardize the initial quantity, we conducted the experiments using 3 mm skin fragments, which is similar to a skin punch for leprosy diagnosis. Following these initial analyses optimized with the porcine model, we proceeded with evaluations of clinical samples using 3 mm biopsy fragments.

**Reviewer 3:** The results are too detailed with regards to the intermediate steps before the final protocol. There should be the performances with regard to the test used in Brazil with the portable machine and the quantstudio on the clinical samples. The authors choose to have two targets, the 16S rDNA and the RLEP repeated element present only on *Mycobacterium leprae* genome. Since this element is repeated 35 to 37 in the genomes, this PCR should be more sensitive with a shorter CT. This is well shown in the analytical sensitivity results. However, can the authors explain why the CT values are similar, or even shorter for the 16S results? (table 3).

**Answer:** We appreciate the reviewer´s comments. The results section was completely rewritten, and the intermediate steps were moved to supplemental materials. Repeatability and reproducibility analyses were based on synthetic DNA (gBlock) containing a single copy of the *16S rRNA* and RLEP genes, so similar Ct scores for the different targets are expected (new S7 TABLE, former Table 3). In the analytical sensitivity analyses (FIGURE 3), we used DNA extracted from purified *M.leprae* cells, which resulted in differences in the number of copies detected for each target, as expected due to the fact that *16S rRNA* is present in a single copy and RLEP has several copies in the bacillus genome.

**Reviewer 3:** Table 4: What is the gold standard for measuring the results of performances of the tests on the clinical specimens? clinical diagnosis?

**Answer:** The gold standard used for determining the clinical outcome of each sample is the clinical evaluation. The information was added in the methods section (line 154, page 7).

**Reviewer 3:**The PPV and NPV are interesting only if we know the prevalence of the disease in the population included in the study. Because it was enriched in leprosy patients, this is not informative.

**Answer:** The reviewer is correct and we appreciate the comment. The information has been removed from the text.

**Reviewer 3:**The protocol designed for simplifying the extraction is interesting but not really applicable in the field since it still requires a biology laboratory. Can the authors also simplify this part by giving only the final results with the best protocol.

**Answer:** The reviewer raises an interesting topic. We believe the designed protocols are applicable in field conditions. In fact, we have been using similar protocols for the detection of Plasmodium spp DNA in low-resource areas in the Amazon region and for the detection of *Mycobacterium tuberculosis* DNA in remote areas of the Brazilian coast. All necessary instruments fit in a regular-size food delivery box, thus facilitating the transportation to the activity areas.

“Can the authors also simplify this part by giving only the final results with the best protocol.”

We believe the reviewer is now referring to simplifying the manuscript’s text. We have rewritten the text and now only the results obtained with the best protocol are shown.

**Minor comments:**

1. Explain better what indeterminate results are, and what is the result given to the clinician then?

**Answer:** Indeterminate results in the portable instrument Q3-Plus refer to qPCR results where only one of the markers (16S rRNA or RLEP) was amplified, which does not indicate a definitive positive or negative result (line 239, page 11). These outcomes are inconclusive and require reassessment or additional testing. In clinical practice, regardless of auxiliary tools used (such as bacilloscopy, histopathology or qCPR), clinical signs observed by the physician take precedence and are considered gold standard. Thus, the clinical evaluation determines whether a patient will be diagnosed and treated for leprosy or not. Parameters that define samples as positive, negative or indeterminate in the analyses performed by the QuantStudio5 instrument were also included in the text (line 262-268, page 11 and 12).

2. The discordant results between Q3 plus and QS5 could be detailed with regard to the gold standard.

**Answer:** We thank the reviewer for the suggestion and have included a paragraph about comparing these results in the discussion (line 510, page 26).

**Conclusions**

**Reviewer 1:** Are the conclusions supported by the data presented? Yes but can be more structured. Are the limitations of analysis clearly described? No limitations stated.

Do the authors discuss how these data can be helpful to advance our understanding of the topic under study? -Is public health relevance addressed? yes

**Reviewer 2:** It is not possible the way the data were shown to evaluate if the conclusions are supported by the data because they should separate and show the Ct values for the MB, PB and OD groups. This would make evaluation of the usefulness of this test clearer.

**Answer:** To facilitate the evaluation, we separated the data for the MB, PB and OD groups according to the reviewer's suggestion (2 and 3 Table), and the Ct values are presented in supplementary tables (S5 and S9).

**Reviewer 3**: The conclusions about a portable test for leprosy diagnosis are not reached yet. The protocol is still quite complicated (see figure 6) for a primary care center. We do not know the Q3-plus apparatus (need to describe shortly its advantages and disadvantages), is it easily found in the international market?

**Answer:** The Q3-Plus instrument is considered portable due to its small dimensions (7x14x8.5 cm) and lightweight (300 g) and is described in details elsewhere (<https://doi.org/10.3390/s18082583>). As shown by Cereda et al. (2018), it performs the same analyses as a standard benchtop equipment with excellent results. The name Q3 reflects its main advantages: qualitative, quantitative, and quick. Additionally, it is user-friendly and requires small sample volumes for analysis (We have added a photo of the Q3-Plus below this comment).

Simplified extraction protocols consist of a denaturing solution, filter paper, paper punch, and elution buffer (extraction module). The portable kit consists of the extraction module, dry bath, Q3-Plus, vortex, mini centrifuge, pipettes, tips, gloves, and all the material necessary for collecting and processing the samples. Therefore, it is easily transported and used in a primary care center. In fact, we have already been using similar portable tests for malaria and tuberculosis in primary care centers in the Amazon and coastal regions of Brazil.

The Q3-Plus instrument is currently sold under the fantasy name Molecular Mouse, by the Italian company Alifax (<https://www.alifax.com/products/molecular-mouse/>). However, and more importantly, the main purpose of the manuscript is to validate the performance of the extraction protocol, since many portable qPCR instruments are now available in the international market, and our simplified extraction protocol could be used in any of them.

qPCR Q3-Plus equipment. The dimensions are 7x14x8.5 cm.

Created by the author.

**Reviewer 3**:The authors could present only their final protocol with the analytical results and performances on the clinical samples. Authors need to explain what brings to the results the analyses of the 16S and the 18S rRNA, which is far too complicated for the field.

**Answer:** The analysis of both targets plays a crucial role as controls. The inclusion of *18S rRNA* detection in the test ensures the presence of human material in the sample, acting as an internal positive control that validates successful collection, extraction, and qPCR steps. The inclusion of the *16S rRNA* detection ensures that the extraction protocol and the qPCR reaction were properly executed, since *16S rRNA* is a single-copy, conserved gene, showcasing the sensitivity of the test (line 532-538, page 27 and 28).

**Editorial and Data Presentation**

1. Table 2 shows the six lysis solutions that were evaluated in the development of a simplified extraction method. Eventually, the optimal extraction method identified was solution 2 (line 290) composed of PBS (366 ul), proteinase K (14.6 ul), urea (146 ul) and nuclease free water (73.4 ul). What would have been ideal would be to show a comparison of the qPCR curves for a known quantity of *M. leprae* cells extracted with these six solutions to show the differences between detecting each of the *M. leprae* targets, 16S rRNA and RLEP. This could have been shown as LOD values obtained from these six extraction solutions.

**Answer:** We agree with the reviewer, but we would like to point the difficulty in accessing this type of sample due to the inability to cultivate *M. leprae* in vitro, in addition to the research group’s lack of access to biological model cultivation (mouse footpads), thus rendering the suggested experiment unfeasible under the current circumstances. However, in order to standardize the initial sample quantity, we used 3 mm fragments of porcine skin to evaluate each lysis solution. Accordingly, 3 mm biopsy samples from patients were collected at the Souza Araújo Clinic.

2. Table 5 shows something along these lines by evaluating the six solutions with the 18S rRNA as the target by qPCR in porcine skin samples but it would have been best to show the two *M. leprae* targets and their Ct scores.

**Answer:** We agree with the reviewer, however using *M. leprae* cells is unfeasible under the current circumstances. Moreover, amplification of naturally-ocurring *M. leprae* targets would be unlikely in porcine skin. For a spike test, we would need a known number of *M. leprae* cells, which is unfeasible, as discussed earlier.

3. It was mentioned in the Discussion in lines 686-694 noted that as is the case with previous PCR and qPCR detection of various targets and biological materials, PB cases show a variable range of sensitivity from 36.4% to 85%. Lines 221-227 detail the numbers of MB and PB patients and other dermatoses (OD) where skin biopsies were extracted, also shown in supplementary Table S1. It is not clear from the Results section in lines 540-555 showing the numbers of samples tested (n = 53) what the positive “agent” is (16S rRNA or RLEP, either or both). The number seems to be an aggregate here but it does not differentiate between MB, PB and OD samples. Ideally, the number of positives should be reported for each separate group for each target since the percentage of positives would likely be higher for MB than PB. Since the BI for the majority of PB cases is 0, it would not be surprising to see that the sensitivity for the qPCR reaction using the NAT Hans kit on the Q3-Plus equipment would be low but this information is also important. Reporting the differences in the sensitivity between these two groups, MB versus PB, would be preferred. It is assumed almost all of the OD cases would be negative for both targets but it is not clear from the presentation of the data if this is the case.

**Answer:** Thank you for the suggestion. The groups were separated according to the clinical form (PB, MB, OD), and analyses of sensitivity, specificity, and accuracy according to each group were included. These data are presented in tables 2 and 3 (line 378 and 459, page 19 and 24). A supplementary table (S5 TABLE) with the Ct scores of each evaluated sample was also included in the material. Samples that were discordant between the equipment and the gold standard (clinical analysis) were discussed. Those samples that were negative in the clinical evaluation (OD samples) but had positive results in the molecular analyses were also included in the discussion.

4. The BI for MB cases in Table S1 ranges from 1-5.75. It is likely and expected that the majority of positive responses for the qPCR test would be positive for MB patients with a BI >2.0 but it would be good to mention this in the Discussion.

**Answer:** Thank you for the suggestion. We analyzed the MB cases and included them in the discussion (line 510, page 26).

5. Two of the OD individuals in Table S1 have a positive BI, line 84 (1.5) and line 102 (4.5). Is this correct and were either of these individuals positive by qPCR?

**Answer:** There was a typing error. The individual in line 84 (1.5) is a PB patient and has an iB 0, and the individual in line 102 (4.5) is MB. The PB individual tested negative by qPCR, while the MB individual tested positive.

**Minor points**

Line 163, “mammals” should probably be “humans”

**Answer:** This target was developed for mammals in general.

Table 2, unclear if the abbreviation PK stands for proteinase K?

**Answer:** Yes, the abbreviation refers to proteinase K. It was added to the table. Thank you.

Line 368, “a.u.” abbreviation not defined

**Answer:** It has been corrected. The abbreviation is arbitrary units of fluorescence. Thank you.

Line 548, “testes” should be “tests”

**Answer:** Thank you. This paragraph was altered.

Line 565, “sensibility” should be “sensitivity”

**Answer:** Thank you. This paragraph was altered.

Line 574, “adjunctive” should be “adjunct”

**Answer:** It has been corrected. Thank you.

**Reviewer 3:** Delete many tables and figures or put them as supplementary data.

**Answer:** Figures and tables have been added to the supplementary material.

**Summary and General Comments**

**Reviewer 1: Minor revision**

A study that is needed, because we need more field friendly DNA extraction and qPCR for leprosy diagnosis. Overall the article would benefit from a better flow, maybe first evaluating the extraction and then the qPCR, now it is quite confusing which samples are used for what and what is analyzed for what. Maybe a flowchart or outline of the steps undertaken that lead to the DNA extraction evaluation and the qPCR evaluation. I think the article can be more concise and better structured.

**Answer:** Thank you for the suggestions. We have created a diagram to clarify the steps and the samples used in each stage of the study. We have included the diagram in the Materials and Methods section (line 169, page 8)(Figure 1). The structure of the article has also been revised according to the reviewers’ suggestions to make it concise. Intermediate steps of the portable equipment optimization have been included in the supplementary material, and in the main text we have objectively described the analysis of the protocol developed for DNA extraction from skin biopsies and the reactions on the portable platform.

line 55: FI is not a worldwide use abbreviation: The common abbreviation is GD G0D: grade-0 disability. G1D: grade-1 disability. G2D: grade-2 disability.

**Answer:** It has been corrected. Thank you.

line78: analytical sensitivity: the analytical sensitivity stays the same, the diagnostic sensitivity changes.

**Answer:** It has been corrected. Thank you.

line 81: abbreviate explanation of NAT and not for first time in line 102.

**Answer:** It has been corrected. Thank you.

line 134-135: copies 16S or RLEP or 18S or all three?

**Answer:** For all three targets. The information has been added to the text.

line 144: double point

**Answer:** It has been corrected. Thank you.

line 145: Is this standard curve used to make the standard curve for the qPCR? Or for evaluating the extraction? For the extraction it is the right choice, for the qPCR not, therefore plasmid DNA or gDNA should be used.

**Answer:** This standard curve with purified DNA from *M.leprae* samples was used to evaluate qPCR (S7 FIGURE). However, a standard curve was also generated using synthetic DNA (gBlock) (S6 FIGURE).

Table 1: What is the Gain? Of what?

**Answer:** The gain is one of the optical parameters responsible for the qPCR experiment readout by the Q3-Plus equipment. It is accountable for the increase in optical signal intensity. Table 1 has been removed from the article because we understand that, as it represents an intermediate step in the optimization of the Q3-Plus, it is not essential for the paper. Following the reviewers’ suggestion, we believe this will contribute to making the text more concise.

Table 2: Not clear in the table from where the skin biopsies come, human or pork? In line 215 3 mm skin biopsies are used, so what is the rationale for the 3 mm skin biopsy?

**Answer:** Table 2 (S2 TABLE) displays the lysis solutions evaluated during the initial phase of the simplified extraction protocol development. Initially, all solutions were tested using swine skin as the experimental model, which were purchased at a local market. Following the identification of the most effective lysis solution, which was solution 2, we proceeded to evaluate using skin biopsies from patients treated at the Souza Araújo Clinic. We included sample identification in the table title. We used 3 mm biopsies because it is the collection methodology currently used in the diagnostic routine at Souza Araújo Outpatient Clinic.

**Reviewer 3:** The paper is far too long and too descriptive of intermediate steps to reach the final version of the protocol. The authors should focus on the performances on the clinical samples detailing the gold standard they choose to interpret their results.

**Answer:** We appreciate the feedback. Changes to the article have been made with the aim of improving its structure and making it more concise.
